# Effects of Channel Thickness on Electrical Performance and Stability of High-Performance InSnO Thin-Film Transistors

**DOI:** 10.3390/membranes11120929

**Published:** 2021-11-26

**Authors:** Qi Li, Junchen Dong, Dedong Han, Yi Wang

**Affiliations:** 1 Institute of Microelectronics, Peking University, Beijing 100871, China; 1901111209@pku.edu.cn (Q.L.); imewangyi@pku.edu.cn (Y.W.); 2 School of Information & Communication Engineering, Beijing Information Science & Technology University, Beijing 100101, China

**Keywords:** ITO TFTs, channel thickness, electrical characteristics, stability

## Abstract

InSnO (ITO) thin-film transistors (TFTs) attract much attention in fields of displays and low-cost integrated circuits (IC). In the present work, we demonstrate the high-performance, robust ITO TFTs that fabricated at process temperature no higher than 100 °C. The influences of channel thickness (t_ITO_, respectively, 6, 9, 12, and 15 nm) on device performance and positive bias stress (PBS) stability of the ITO TFTs are examined. We found that content of oxygen defects positively correlates with t_ITO_, leading to increases of both trap states as well as carrier concentration and synthetically determining electrical properties of the ITO TFTs. Interestingly, the ITO TFTs with a t_ITO_ of 9 nm exhibit the best performance and PBS stability, and typical electrical properties include a field-effect mobility (µ_FE_) of 37.69 cm^2^/Vs, a V_on_ of −2.3 V, a SS of 167.49 mV/decade, and an on–off current ratio over 10^7^. This work paves the way for practical application of the ITO TFTs.

## 1. Introduction

Metal-oxide thin-film transistors (TFTs) are recognized as a promising alternative to conventional hydrogenated amorphous silicon (a-Si:H) TFTs because of high performance, feasibility for flexible display, and good process compatibility with the a-Si:H TFTs [1,2,3]. Despite of these advantages, mobility and stability of metal-oxide TFTs need to be further improved to meet the increasing demands for advanced displays of fast frame rate, ultrahigh resolution, and large area [4,5,6].

InSnO (ITO) is a kind of highly conductive material with a wide bandgap (3.5~4.3 eV) and high optical transmittance (~90%), which generally serves as transparent electrodes in electron devices [7,8,9]. High conductivity of the ITO films origins from a facile pathway for electron conduction that is introduced by a large overlap of 5 s orbits of In and Sn elements [10]. Recently, ITO has been utilized as channel material of TFTs. Park et al. explored high-pressure annealing (HPA) treated ITO TFT with a saturation mobility (µ_sat_) of 25.8 cm^2^/Vs [11]. Liang et al. demonstrated ITO TFTs with a high µ_sat_ of 34.9 cm^2^/Vs as well as excellent stability [12]. Thereby, the ITO TFTs show immense potential in the field of display. However, the underlying mechanisms of ITO thickness (t_ITO_) on device performance of the ITO TFT are still not fully understood.

In this work, ITO TFTs with a t_ITO_ of 6, 9, 12, and 15 nm are fabricated. To analyze the dependence of device performance and stability on channel thickness, a systematic study on ITO films and ITO TFTs is conducted. The ITO TFTs with a t_ITO_ of 9 nm exhibit the best performance, the typical properties include a filed-effect mobility (µ_FE_) of 37.69 cm^2^/Vs, a turn-on voltage (V_on_) of −2.3 V, an on–off current ratio (I_on_/I_off_) over 10^7^, and a subthreshold swing (SS) of 167.49 mV/decade. Moreover, the ITO TFTs show excellent positive bias stress (PBS) stability, and threshold voltage shift (∆V_TH_) is 0.46 V under 1000 s, +1 MV/cm stress.

## 2. Experiment

### 2.1. Fabrication of ITO TFTs

**Figure 1:**
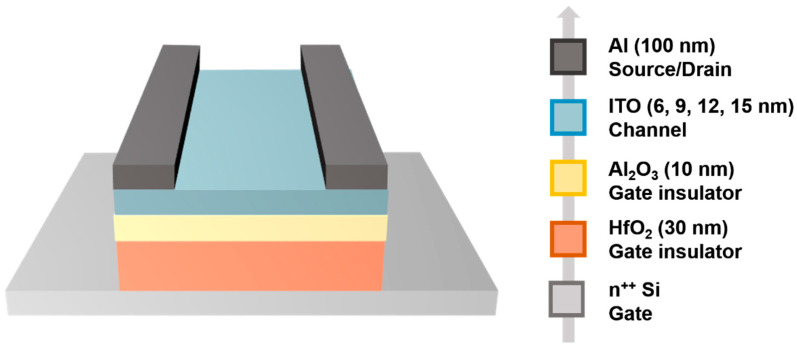
Schematic device structure and device fabrication procedure of ITO TFTs.

Figure 1 shows schematic structure of the ITO TFTs. Feature size of devices is width/length (W/L) = 100 μm/100 μm. Firstly, a heavily doped Si substrate was ultrasonic cleaned in acetone, alcohol, and deionized water, respectively. The Si substrate also acts as gate electrode. Next, a 30-nm HfO_2_ dielectric layer was deposited by sputtering process at room temperature. Then, a 10-nm Al_2_O_3_ dielectric layer was deposited by atomic layer deposition (ALD) process at 100 °C. Subsequently, an ITO channel layer was deposited by sputtering process, and the sputtering process was performed in Ar/O_2_ gas mixture (Ar/O_2_ flux ratio = 80/20) with a power of 70 W and a pressure of 1 Pa. Finally, a 100-nm Al source/drain electrode was deposited by sputtering process at room temperature. The ITO TFTs were patterned by lithography and lift-off processes. Before we measured device performance, the ITO TFTs were thermally annealed in vacuum at 100 °C for 1 h.

### 2.2. Characterization of ITO TFTs and ITO Films

Current-voltage (I-V) curves of the TFTs were characterized in dark at room temperature using a semiconductor parameter analyzer (Agilent B1500A). Capacitance properties of the metal–insulator–semiconductor (MIS) structure were measured using a semiconductor characterization system (Keithley 4200).

Microstructure of the ITO films were characterized by X-ray diffraction (XRD, Rigaku D/MAX 2000) and transmission electron microscopy (TEM, FEI Tecnai F20). Surface morphology of the ITO films were characterized by atomic force microscopy (AFM, Bruker Dimension Icon) and scanning electron microscope (SEM, FEI Helios Nanolab G3 CX). Chemical properties of the ITO thin films were examined using X-ray photoelectron spectroscopy (XPS, Axis Supra).

## 3. Results and Discussion

### 3.1. Material Characterization of ITO Films

**Figure 2:**
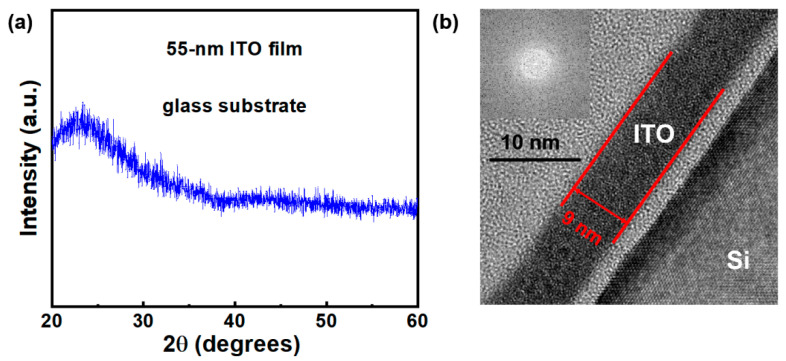
(**a**) XRD spectrum of ITO film on glass substrate. Film thickness is 55 nm. (**b**) TEM image and FFT image of ITO film.

Figure 2a exhibits XRD spectrum of the ITO films. To accurately characterized diffraction peaks, the ITO films with a thickness of 55 nm were prepared on glass substrate. The obtained diffraction patterns contain only two broad peaks at approximately 23° and 45°, originating from the glass substrates [13]; this suggests that the ITO films have an amorphous phase. Normally, the amorphous phase of the ITO active layer is beneficial to the uniformity and stability of the ITO TFTs. In order to gain further insight into lattice structure of the ITO film, TEM measurement was performed, as shown in Figure 2b. It is observed that thickness of the ITO film is about 9 nm. No local crystalline grain can be observed. We performed real-time fast Fourier transform (FFT) of the ITO films, as shown in inset of Figure 2b. The FFT image exhibits amorphous diffraction pattern; thus, the lattice structure of the ITO film is definitely amorphous.

**Figure 3:**
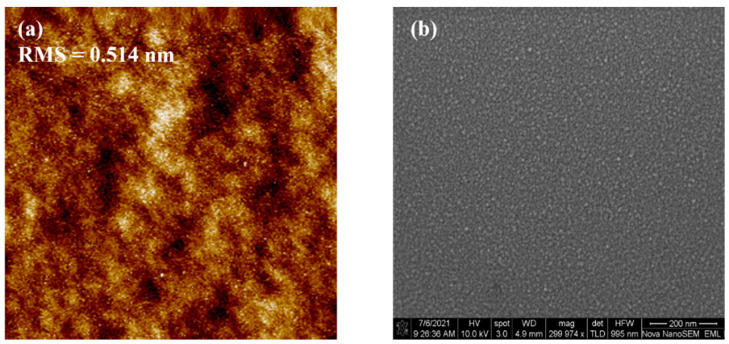
(**a**) AFM image of the ITO film. Scanning area is 5 µm × 5 µm. (**b**) SEM image of ITO film. Film thickness is 9 nm.

Figure 3a depicts AFM image of the ITO film, and the scanning area is set as 5 µm × 5 µm. Remarkably, the ITO film exhibits extremely flat surface morphology, and root-mean-square (RMS) roughness is 0.514 nm. Figure 3b shows SEM image of the ITO film. We can see that the local grains compactly and uniformly arrange with each other. The AFM and SEM validate smooth surface of the ITO films, which takes effect in reducing surface scattering and enhancing device performance of the ITO TFTs [14].

### 3.2. Electrical Characteristics of ITO TFTs

**Figure 4:**
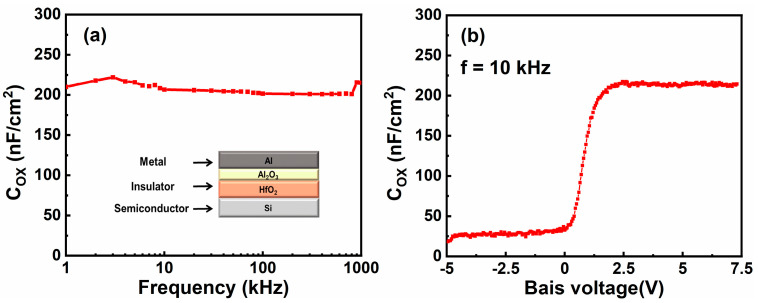
(**a**) C–F curve of the MIS structure. (**b**) C–V curve of the MIS structure. Frequency is 10 KHz.

A MIS structure of Al-HfO_2_/Al_2_O_3_-Si was fabricated to determine capacitance properties. Fabrication process of the HfO_2_/Al_2_O_3_ bilayer dielectric is mentioned above. The Si substrate is n-type, lightly doped. Figure 4a exhibits the capacitance–frequency (C–F) curve of the MIS structure. Capacitance per unit area (C_OX_) maintains a value of about 210 nF/cm^2^ with frequency from 1 KHz to 1 MHz, implying high film quality of the HfO_2_/Al_2_O_3_ bilayer dielectric. Figure 4b exhibits capacitance–voltage (C–V) curve of the MIS structure at a frequency of 10 KHz. The C–V curve exhibits typically high-frequency capacitance property. C_OX_ of the MIS structure is 214.55 nF/cm^2^ when voltage is 5 V, which is consistent with the C–F curve.

**Figure 5:**
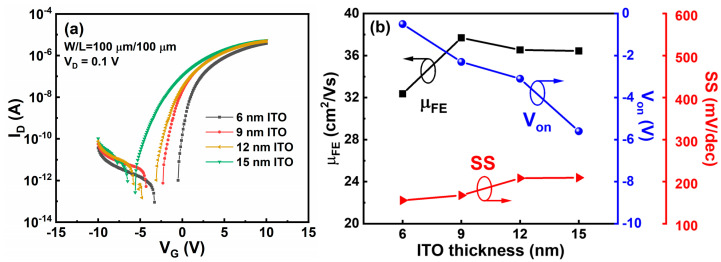
(**a**) Transfer curves of ITO TFTs. V_D_ = 0.1 V. (**b**) Electrical parameters of ITO TFTs, including μ_FE_, V_on_, and SS.

To examine effects of t_ITO_ on device performance of the ITO TFTs, drain current–gate voltage (I_D_–V_G_) curves were measured, as shown in Figure 5a. All of the devices show an on-state current (I_on_) higher than 1 μA and an I_on_/I_off_ over 10^7^. Electrical properties of μ_FE_, V_on_, and SS are extracted, as shown in Figure 5b. Here, µ_FE_ is calculated using the following equation:

(1) $$\mu_{\mathrm{FE}}=\frac{\partial I_{D}}{\partial V_{G}}\times \frac{L}{{\mathrm{WC}}_{\mathrm{OX}}V_{D}}$$

where L and W are channel length and channel width, respectively [15]. V_D_ is drain voltage and is set as 0.1 V. SS is extracted from the linear part of a plot of the log (I_D_) versus V_G_, using SS = dV_G_/dlogI_D_ [16]. V_on_ is defined as the gate voltage at which I_D_ starts to monotonically increase [17]. We found that all of the devices show a μ_FE_ larger than 30 cm^2^/Vs. Notably, μ_FE_ reaches a peak value when t_ITO_ is 9 nm. Moreover, V_on_ and SS present a negative and a positive correlation with t_ITO_, and significant degeneration of V_on_ and SS occurs as t_ITO_ increases to 12 and 15 nm, which results from large number of free electrons and higher sheet trap density in the channel layer [18,19]. Consequently, the ITO TFTs with 9-nm ITO active layer show the best electrical properties, such as a μ_FE_ of 37.69 cm^2^/Vs, a V_on_ of −2.3 V, and a SS of 167.49 mV/decade.

**Figure 6:**
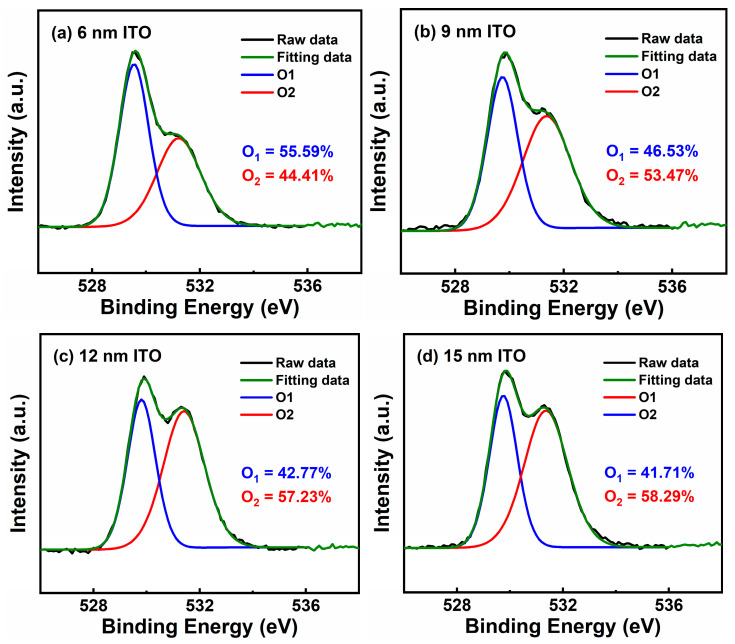
Deconvolution of the O 1 s XPS spectrum of ITO films with thickness of (**a**) 6 nm, (**b**) 9 nm, (**c**) 12 nm, and (**d**) 15 nm.

In order to deeply understand the physical mechanism behind the electrical performances of ITO TFTs, we characterized the ITO films with different thickness by XPS, as shown in Figure 6. The binding energy (BE) was calibrated by the standard C 1 s line at 284.80 eV [20]. The O 1 s peak is deconvoluted into two components: O_1_ peak at around 529.7 eV and O_2_ peak at around 531.3 eV, which can be regarded as metal–oxygen lattice and oxygen defects (oxygen vacancies and chemisorbed oxygen element), respectively [21]. Here, the ratio of oxygen defects is defined as the peak area ratio of O_2/_(O_1_ + O_2_) and is positively correlated with t_ITO_. The oxygen defects can serve as interface traps; therefore, the SS is deteriorated with increasing t_ITO_ due to the increase of oxygen defects [22,23]. It is known that the content of oxygen vacancies, which normally acts as a shallow donor in oxide semiconductor, directly affects the carrier concentration of ITO films [1]. Consequently, the V_on_ negatively shifts with increasing t_ITO_ and the μ_FE_ increases with t_ITO_ increasing from 6 to 9 nm. However, similar to other impurity dopants, more oxygen defects can induce more ionized impurity scattering, which possibly results in the degradation of μ_FE_ with t_ITO_ increasing from 9 to 15 nm [24].

**Figure 7:**
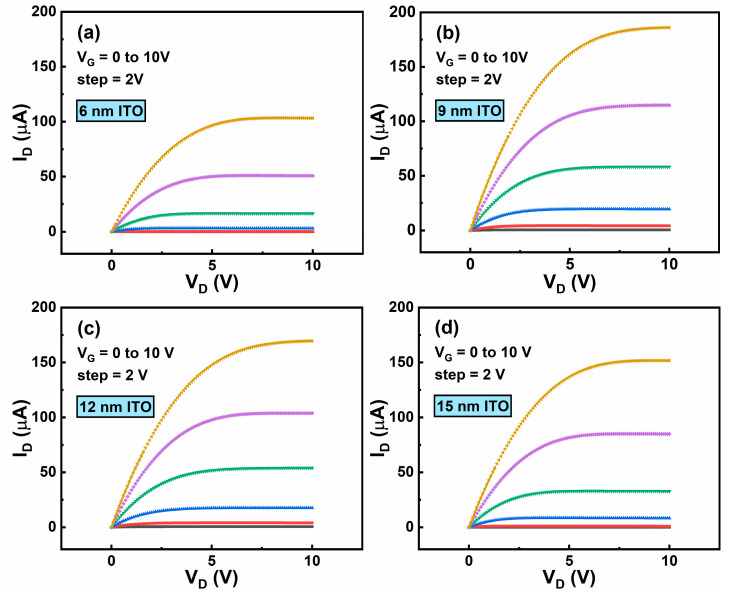
Output characteristics of ITO TFTs with t_ITO_ of (**a**) 6 nm, (**b**) 9 nm, (**c**) 12 nm, and (**d**) 15 nm.

Drain current–drain voltage (I_D_-V_D_) curves of the ITO TFTs are measured, as shown in Figure 7a–d. All the I_D_–V_D_ curves show apparent linear and saturation region. As t_ITO_ increases from 6 to 15 nm, the saturation current increases first and then decreases, and the maximum value of 184 µA is observed when t_ITO_ is 9 nm. Additionally, there is no obvious current crowding phenomenon for all the ITO TFTs, indicating good Ohmic contact between ITO channel layer and Al source/drain electrodes [25].

**Figure 8:**
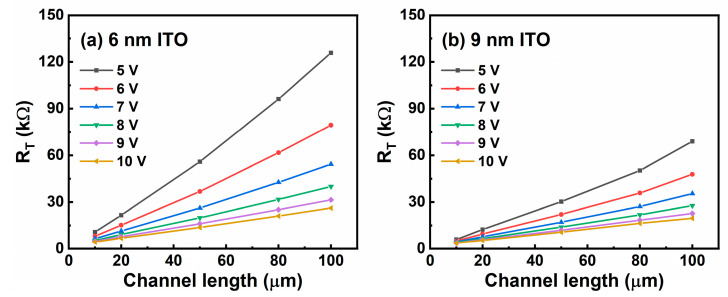

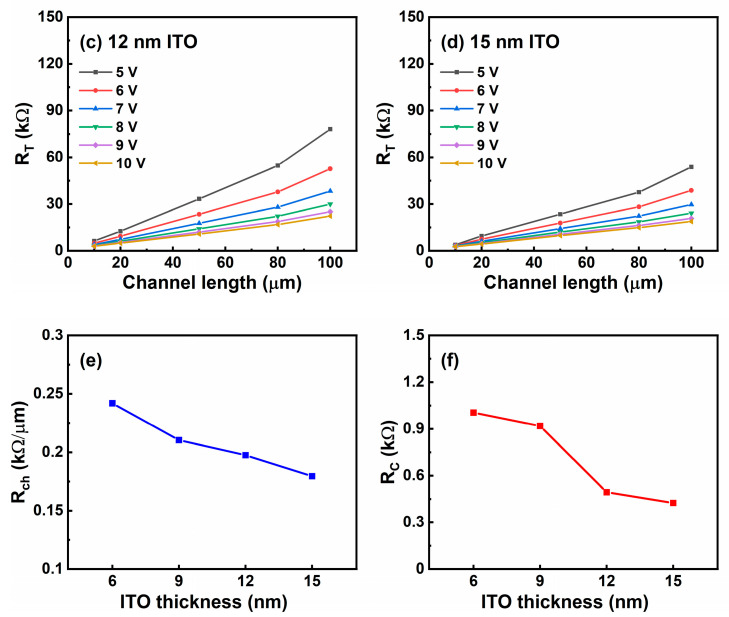
Total resistance (R_T_) of ITO TFTs with t_ITO_ of (**a**) 6 nm, (**b**) 9 nm, (**c**) 12 nm, and (**d**) 15 nm. (**e**) R_ch_ and (**f**) R_C_ as a function of t_ITO_.

In order to comprehensively analyze contact property between ITO channel layer and Al source/drain electrode, we extract contact resistance (R_C_) based on transfer line method (TLM). Total resistance (R_T_) of the TFTs at on-state is expressed as R_T_ = R_ch_L + 2R_C_, where R_ch_ and L represent channel resistance per unit length and channel length, respectively [26]. R_T_ versus L at different V_G_ for the ITO TFTs are shown in Figure 8a–d. By applying linear fitting, we obtain R_ch_ and R_C_ of the ITO TFTs, as shown in Figure 8e,f. Significantly, both R_ch_ and R_C_ present a negative correlation with t_ITO_, which attributes to the increasing amount of conductive electrons in the ITO channel layers. Thereby, contact property can be enhanced by increasing t_ITO_.

In the aspect of contact property, we found that increasing t_ITO_ plays a role in enhancing contact property of the ITO TFTs. Commonly, a preferable contact property is desirable for high-performance metal–oxide TFTs. However, we verify that V_on_ and SS dramatically degrade when t_ITO_ increases to 12 and 15 nm (Figure 5b). μ_FE_ and saturation current achieve the maximum value when t_ITO_ is 9 nm. That is to say, an optimal t_ITO_ is 9 nm in this work.

**Figure 9:**
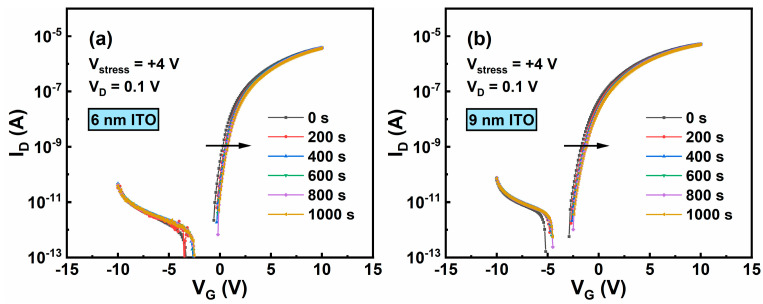

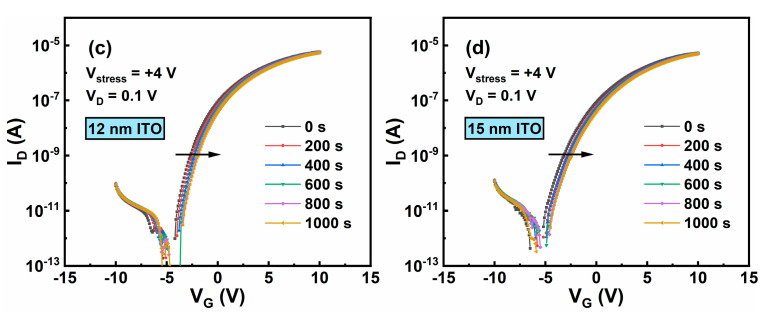
PBS (V_G_ = +4 V) of ITO TFTs with t_ITO_ of (**a**) 6 nm, (**b**) 9 nm, (**c**) 12 nm, and (**d**) 15 nm.

**Figure 10:**
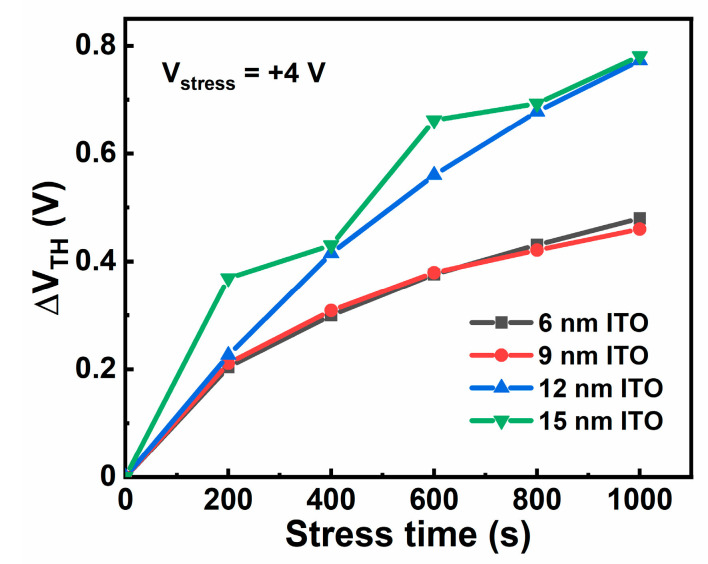
∆V_TH_ for the ITO TFTs with different t_ITO_ under PBS at different stress times.

Finally, PBS stability of the ITO TFTs are measured, as shown in Figure 9a–d. Stress conditions are as follow: the stress voltage applied on the gate electrode is +4 V with the source and drain electrodes grounded, and the stress duration is 1000 s. Figure 10 presents ΔV_TH_ of the ITO TFTs with different t_ITO_ under PBS at different stress times. In general, the devices show improved stability under PBS with reducing t_ITO_, which is consistent with the previously reported results [27,28,29]. One possible mechanism related to PBS is the oxygen vacancy model [30]. As shown in Figure 6, for ITO TFT with a larger t_ITO_, there are more oxygen vacancy defects, causing a larger positive ∆V_TH_.

## 4. Conclusions

In conclusion, high-performance, robust ITO TFTs are fabricated at a maximum process temperature of 100 °C. We investigate the effects of t_ITO_ on the electrical characteristics and PBS stability of ITO TFTs with t_ITO_ of 6, 9, 12, and 15 nm. We found that content of oxygen defects positively correlates with tITO, leading to increase of both trap states as well as carrier concentration, and synthetically determining electrical properties of the ITO TFTs. Interestingly, the devices with a 9-nm ITO thickness show the best performance with a large µ_FE_ of 37.69 cm^2^/Vs, a high I_on_/I_off_ over 10^7^, a reasonable V_on_ of −2.3 V, and a steep SS of 167.49 mV/decade. Moreover, the device exhibits preferable stability under PBS (∆V_TH_ = 0.46 V). Overall, our ITO TFTs show great potential in next-generation displays.

## Data Availability

The data that support the findings of this study are available from the corresponding author upon reasonable request.
